# A mini review of the pathogenesis of acute rheumatic fever and rheumatic heart disease

**DOI:** 10.3389/fcimb.2025.1447149

**Published:** 2025-04-10

**Authors:** Shuting Zhuang, Danchun Guo, Dingle Yu

**Affiliations:** Department of Respiration, Shenzhen Children’s Hospital, Shenzhen Univesity, Shantou University Medical College, Shenzhen, China

**Keywords:** rheumatic fever, rheumatic heart disease, group A *Streptococcus* (GAS), pathogenesis, vaccine

## Abstract

Acute rheumatic fever (ARF) is an autoimmune disease caused by group A streptococcal infection. Recurrent episodes of ARF can lead to rheumatic heart disease (RHD), which is the leading cause of cardiovascular mortality in children worldwide, especially in low- and middle-income countries. Investigations into the etiology of ARF and RHD constitute a crucial milestone in the advancement of both preventive measures and therapeutic interventions. The purpose of this mini review is to delineate the etiology and pathophysiological mechanisms underlying ARF and RHD. Selective searches were conducted in PubMed to retrieve literature published between 1968 and 2024, employing key terms such as “acute rheumatic fever”, “rheumatic heart disease”, “group A *Streptococcus*”, “streptococcal pharyngitis”, “pathogenesis”, and “pathophysiology”. The pathogenesis of infections caused by group A streptococci, and their effects on ARF and RHD, have been thoroughly examined. A central hypothesis is that autoimmune responses are triggered by molecular mimicry, but alternate pathogenic mechanisms are continuously being explored. There is an urgent need for high-quality research that can inform efforts aimed at decreasing the occurrence of ARF and halting the advancement of RHD, which requires researchers to understand its causes and to develop appropriate preventive and therapeutic programs.

## Introduction

Acute rheumatic fever (ARF) is an immune disorder triggered by repeated infections with group A *Streptococcus* (GAS) (Yu et al., 2023, Yu et al., 2024). Recurrent ARF can lead to rheumatic heart disease (RHD) (Arvind and Ramakrishnan, 2020; Oliver et al., 2021; Karthikeyan and Guilherme, 2018; Middleton et al., 2022; Armitage et al., 2024; Rivera-Hernandez et al., 2019). Human GAS infection is a multifaceted process involving both host and bacterial factors (Brouwer et al., 2023). From an epidemiological perspective, the global epidemiological profile of GAS is variable. The emergence of new GAS clones is often associated with the acquisition of new pathogenicity or resistance determinants (Brouwer et al., 2023). From an immunological perspective, repeated infections with GAS reduce the tolerance of the host immune system and trigger an autoimmune response.

## Diagnostic criteria for ARF

ARF is a delayed, non-purulent sequela of a GAS pharyngeal infection (Chakravarty et al., 2014; Pilapitiya et al., 2021). After experiencing an initial bout of pharyngitis, an incubation period of one to five weeks precedes the emergence of initial signs or symptoms associated with ARF. Notably, while pharyngitis is a known trigger for both ARF and RHD, other superficial ailments, including scarlet fever, impetigo, and obstructive sleep apnea hypopnea syndrome, also contribute to the occurrence of these conditions (Chakravarty et al., 2014; Liang et al., 2023; Carapetis et al., 2005; Chiou et al., 2004; Barth et al., 2022; Viciani et al., 2016). A diagnosis of ARF is determined by applying clinical criteria (Jones criteria 2015) while excluding other potential differential diagnoses (Carapetis et al., 2016; Gewitz et al., 2015). Clinical criteria are divided into primary and secondary manifestations. Primary manifestations include carditis, arthritis, chorea, subcutaneous nodules, and erythema marginatum. Secondary symptoms include fever, arthralgia, elevated sedimentation rate, increased C-reactive protein, and increased PR interval (Chakravarty et al., 2014; Carapetis et al., 2016). For a confirmed diagnosis of ARF, a patient must exhibit two major symptoms or one major symptom with at least two minor symptoms (Chakravarty et al., 2014). A history of GAS infection must also be demonstrated, which can usually be accomplished by streptococcal serology. Elevated antistreptolysin O titer, elevated anti-DNase B titer, or a positive culture for GAS in the pharynx is considered supportive evidence of GAS infection (Arvind and Ramakrishnan, 2020). It should be noted that damage to the heart valves is a chronic and progressive condition that may ultimately result in heart failure (Kayima and Kaddumukasa, 2024). These symptoms may not become apparent until months after the onset of the causative streptococcal infection, when other symptoms may be absent and streptococcal serologic tests normal.

## Susceptibility to ARF and RHD

ARF may be an autoimmune response to GAS pharyngeal infection, especially in genetically susceptible populations, and evidence supports that a molecular mimetic mechanism likely mediates this response. Approximately 0.30–3.00% of patients with GAS pharyngitis may develop ARF, the probability of which is influenced by the host’s genetic susceptibility, recurrent infections, and the virulence level of the infecting strain (Karthikeyan and Guilherme, 2018; Brouwer et al., 2023; Liang et al., 2023; Carapetis et al., 2016; de Crombrugghe et al., 2020; Passos et al., 2021). Host genetic susceptibility is mainly caused by HLA class II alleles DR and DQ, B-cell antigen D8/17, and genetic polymorphisms for tumor necrosis factor–α (TNF-α), interleukin, or transforming growth factor-β1 (TGF-β1), as well as significantly fewer germinal center (GC) -T follicular helper (Tfh) cells (Arvind and Ramakrishnan, 2020; Chakravarty et al., 2014; Guilherme and Kalil, 2010; Bryant et al., 2009; Dan et al., 2019; Crotty, 2019). Included among these, HLA class II alleles associated with RF/RHD susceptibility vary by region (Guilherme et al., 2018). The mechanism of repeated infection is termed “immune priming,” in which repeated infection with GAS renders the immune system intolerant and results in an autoimmune response (Dan et al., 2019). In this process, virulence factors involved in disease progression include those that are surface-bound as well as secreted (Brouwer et al., 2023) (**Table 1**). These virulence factors act through the following mechanisms: promotion of colonization, evasion of host immune response, induction of inflammation and virulence enhancement. As a result, they play a pivotal role in the pathology of ARF. In a word, these factors play a very important role in the progression of autoimmune reactions, especially in aggressive diseases. Understanding the pathophysiological mechanisms of ARF provides a basis for future research and treatment. One example of its application is the classification of GAS strains responsible for rheumatic fever, which is based on the encoding sequence of the 5’ end of the M protein gene (*emm*) (Armitage et al., 2024; Brouwer et al., 2023), an important virulence factor known to trigger autoimmune responses (Lacey et al., 2024; Yu et al., 2021). A total of 261 different emm-types have been identified (Brouwer et al., 2023; Walker et al., 2014; Smeesters et al., 2023). Genotypes associated with ARF include *emm*1, 3, 5, 6, 14, 18, 19, 24, 27, and 29 (de Crombrugghe et al., 2020; Pornrattanarungsi et al., 2022), and genotypes associated with RHD include *emm*1, 5, 18, 16, and 3 (Pornrattanarungsi et al., 2022). In addition to *emm* genotypes, other factors that may influence susceptibility include genetic factors, hypersensitivity reactions, circulating immune complexes, toxic effects of streptococcal preparations, streptococcal adherence, and socioeconomic and nutritional factors (Brouwer et al., 2023; Kaplan, 1979).

**Table 1:** Virulence factors of GAS.

GAS Virulence Factors	Name	Postulated role in pathogenesis	Ref.
Surface-bound virulence factors	M protein	Promotion of colonization and virulence enhancement by binding of various host factors, binding to epithelial cell receptors, inhibition of neutrophil phagocytosis, and restriction of the complement system	(McMillan et al., 2013)
	Hyaluronic acid capsules	Resistance to the complement system and promotion of GAS adhesion to host cells and enhancement of GAS colonization	(Wessels, 2019; Cywes and Wessels, 2001)
	S protein	Promotion of immune escape by binding erythrocytes to the GAS cell surface	(Wierzbicki et al., 2019)
Secreted virulence factors	Deoxyribonucleases (DNases)	Enhancement of bacterial evasion of the innate immune response by degrading the DNA framework of the Neutrophil Extracellular Trap (NET)	(Remmington and Turner, 2018)
	streptokinase	Evasion of host innate immune response degrades exogenous histones by activation of fibrinogen	(Nitzsche et al., 2016)
	immunoglobulin degrading enzyme of *S. pyogenes*	Immune evasion by specific cleavage of conserved N-glycans on IgG antibodies	(Vincents et al., 2004; Agniswamy et al., 2004; Naegeli et al., 2019)
	streptococcal pyrogenic exotoxin B(SpeB)	Immune evasion by cleavage of the ligand proteins E-cadherin, occludin allowing bacteria to cross the epithelial barrier	(Sumitomo et al., 2013)
	streptococcal lysozyme streptolysins S and O(SLS and SLO)	Induction of inflammation by activation of host inflammatory vesicles through pore formation induces IL-1b production	(Richter et al., 2021)
	NAD glycohydrolase(NADase)	Enhanced virulence by binding of NADase to SLO stabilizes both toxins	(Velarde et al., 2017)
	Hyaluronidase	Bacterial proliferation by egradation of hyaluronic acid in host tissues and enhanced virulence by increased concentration and stability of SLO	(Starr and Engleberg, 2006)
	C5a peptidase (SCPA)	Migration of phagocytes to the infected area by specific degradation of C5a and weakening of the immune response by activation of phagocyte-secreted neutrophils.	(Fan et al., 2024; Ji et al., 1998)

## Pathogenesis of ARF and RHD

### Molecular mimicry

Molecular mimicry refers to the sharing of antibodies or T-cell epitopes between the host and a microbe. In GAS, the cross-reactive antigen is a molecular structure that resembles host molecules and initiates an autoimmune response against host tissues (such as the heart) during both infection and immunity (Cunningham, 2019). This causes autoimmune GAS sequelae such as ARF and RHD (Jiee et al., 2024). The widely accepted hypothesis is that the alpha helical coil structure of M-protein in GAS triggers an immune response that may cross-react with other coiled proteins in the body such as cardiac myosin, laminin, and myosin.

### Types of molecular mimicry

Molecular mimicry was originally defined as the presence of identical amino acid sequences between host and bacterial antigens (Cunningham, 2019; Fujinami and Oldstone, 1985; Fujinami et al., 1983; Schwimmbeck and Oldstone, 1989). Subsequent studies using human monoclonal antibodies have since identified other types of molecular mimicry. One type involves the recognition of similar structures by antibodies. For instance, the M proteins of streptococci bear a striking resemblance to the alpha-helical coiled-coil structures of host proteins such as myosins, keratins, poikilins, and laminins. The common regions of these molecules share 40% or less identity, and the cross-reactive sites are not completely identical (Cunningham, 2019; Cunningham et al., 1997). Studies of monoclonal antibodies derived from RHD have identified cross-reactive antibodies that target GAS, group A glycans, N-acetyl-β-D-glucosamine(GlcNAc), heart valve endothelium, laminin, and key epitopes of the stratified basement membranes (Chakravarty et al., 2014; Faé et al., 2006; Galvin et al., 2000; Cunningham et al., 1989; Dale and Beachey, 1985). Furthermore, reactivity of streptococcal M proteins and myocardial myosin was observed between T cells isolated from peripheral blood and rheumatic heart valves. Some studies have indicated that the periodicity of the seven amino acid residues common to group A streptococcal M proteins is identical to that of proteins such as myosin, desmin, poikilodin and keratin (Cunningham, 2019; Manjula et al., 1985; Phillips et al., 1981; Dale and Beachey, 1986).

Another type of molecular mimicry may contribute to immunological cross-reactivity between diverse molecules, such as DNA and proteins or carbohydrates and peptides (Cunningham et al., 1989; Putterman and Diamond, 1998). Antibodies induced by group A carbohydrate epitope GlcNAc react with human cardiac proteins, including myosin and laminin, and are capable of causing humoral immune damage (Cunningham, 2019, Cunningham, 2012). It is important to note that elevated antibody responses against group A carbohydrates are associated with unfavorable clinical outcomes in patients with heart valve disease (Dudding and Ayoub, 1968). Goldstein et al. demonstrated that glycoproteins present in heart valves possess N-acetylglucosamine, leading to the hypothesis that GAS may be responsible for triggering immune cross-reactivity with heart valves (Goldstein et al., 1967). Moreover, recent studies have demonstrated that immunoglobulin G2 (IgG2) subclass autoantibodies, particularly those directed against the GlcNAc epitope, are instrumental in the deposition of RHD heart valve tissue. This class of antibodies is particularly active in response to GAS infections, and they appear to play an important role in the early stages of the disease. They may therefore serve as biomarkers to predict the risk of autoimmune responses following GAS infections (Kirvan et al., 2022). This type of immunological cross-reactivity is based on the existence of an epitope that is shared by two distinct proteins. This epitope can be comprised of an identical or homologous amino acid sequence, or it can arise from two chemically diverse structures.

### Other pathogenic mechanisms

Although the molecular modeling theory is widely accepted as a means of elucidating the pathogenesis of ARF/RHD, mechanisms other than molecular mimicry have been proposed to explain the pathogenesis (**Table 2**). For example, Dr. Lukomski posits that streptococcal collagen-like (Scl) proteins contributes to streptococcal colonization and pathogenesis in humans and animals (Lukomski et al., 2017; Lukomski and McNitt, 2020). It has been postulated that the response to these antibodies against Scl proteins and human collagen is not related to molecular mimicry, although they are present in ARF patients. Karthikeyan and Guilherme put forth the hypothesis that collagen antibodies are produced by epitope diffusion following initial valve injury (Karthikeyan and Guilherme, 2018). Pilapitiya and his colleagues proposed that there is no molecular mimicry between the Scl proteins of streptococcal bacteria and human collagen (Pilapitiya et al., 2021). One hypothesis posits that a sustained disparity between Th_17_ cells and regulatory T cells (Tregs) may disrupt immune tolerance, thereby elevating the susceptibility of the host to disease following streptococcal infection (Wang et al., 2010). The mouse nasopharyngeal-associated lymphoid tissue (NALT) colonization model is regarded as an optimal model for investigating *Streptococcus pyogenes* colonization in the airway (Park et al., 2003, Park et al., 2004). In this process, NALT plays a key role in antigen uptake by promoting the production of Th_17_ cells and their secretion of antibodies, which in turn activate the mucosal immune response (Chen et al, 2016). TGF-β1 is regarded as a pivotal signaling molecule that facilitates the differentiation of Tregs and Th_17_ cells. GAS infection induces the production of TGF-β1, thereby promoting the predominant TGF-β1-dependent differentiation of Th_17_ cells (Wang et al., 2010). Furthermore, evidence indicates that Prothymosin Alpha plays a pivotal role in the pathological immune response of CD8^+^ T-cells regulated by estradiol receptor alpha (ERα), as well as in the process of recognition of type I collagen, in the context of rheumatic heart disease (Passos et al., 2022).

**Table 2:** Pathogenesis of ARF and RHD.

Pathogenesis		Function	Advantages	Disadvantages	Ref.
Molecular mimicry	Amino acid sequence similarity	The periodicity of the seven amino acid residues common to group A streptococcal M proteins is identical to that of proteins such as myosin, desmin, poikilodin, and keratin, which can trigger cross-reactivity leading to humoral immune damage and valvular lesions.	Experimental evidence is sufficient to directly explain cross-immune reactivity, providing a key theoretical basis for therapeutic guidance and vaccine development.	The challenge of using animal models to replicate human specificity and fully elucidate pathogenesis is of great interest.	(Cunningham, 2019)
	Antibody recognition of similar structures				
	Cross-reactivity between different molecules				
Abnormal immune system response	T-cell imbalance	A sustained disparity between Th_17_ cells and Tregs may disrupt immune tolerance, thereby elevating the susceptibility of the host to disease following streptococcal infection.	Emphasis on the critical role of the immune system and the inflammatory response in disease, as well as the potential for multi-targeted interventions.	Mechanisms are characterized by their complexity and are subject to interaction with other mechanisms.	(Wang et al., 2010)
	Dysregulation of cytokine signaling	TGF-β_1_ production activates the IL-1β-GM-CSF axis, promoting inflammation and T-cell migration to the heart.			(Kim et al., 2018)
	Inflammatory vesicle activation	NLRP3 Inflammatory vesicles induce pro-inflammatory cytokines that activate CD4^+^ T cells, worsening the inflammatory response.			(Kong et al., 2022)
Pathogenesis		Function	Advantages	Disadvantages	Ref.
Abnormal immune system response	Chemokine action	CXCL9, also known as Mig, has been shown to promote progression of ARF dysplasia to RHD.	Emphasis on the critical role of the immune system and the inflammatory response in disease, as well as the potential for multi-targeted interventions.	Mechanisms are characterized by their complexity and are subject to interaction with other mechanisms.	(Faé et al., 2013)
	Macrophage and adhesion molecule pathways	M1 Macrophages mediate inflammatory infiltration and valvular fibrosis through the VLA4/VCAM-1 pathway.			(Xian et al., 2024)
	Epigenetic modifications	In the early stages of GAS infection, TNF-α and IL-6 have been seen to trigger epigenetic changes in heart valve cells and to boost pro-fibrotic genes, such as those in the TGF-β1 signaling pathway.			(Karthikeyan et al., 2020)
	IgG_2_ antibody accumulation	In recurrent infections, IgG_2_ accumulation activates valve endothelial inflammation and promotes Th_1_ and Th_17_ attack.			(Kirvan et al., 2022)
	MAIT cell activation	MAIT cells are highly activated to release IFNγ, IL-1β, IL-2 and TNF, which are involved in pharyngitis, invasive GAS disease and ARF.			(Brouwer et al., 2023; Guilherme et al., 2004)
	Dysbiosis	Dysbiosis undermines immune tolerance, causes systemic immune dysregulation and enhances pro-inflammatory responses.			(Nagarajan et al., 2023)
Other pathogenic mechanisms	Autoimmunity to collagen	Scl proteins induce collagen antibodies by epitope diffusion and are associated with valve damage.	The provision of novel perspectives on pathogenesis and new directions for treatment.	The extant evidence for this subject is limited, and the relationship with molecular modeling is unclear.	(Lukomski and McNitt, 2020)

### Involvement of the immune system

A GAS infection initiates a host inflammatory response and immune system activation, which in most cases facilitates the clearance of the infection. However, in some susceptible individuals, an aberrant immune system response occurs, leading to the development of autoimmune diseases such as ARF and RHD. The latest research indicates that immune cells and inflammatory cytokines have a dual function in the pathological process of disease, acting both as promoters and regulators (Nagarajan et al., 2023). Additionally, Because GAS is a human pathogen, animal models of ARF and RHD often fail to replicate essential characteristics of the pathophysiology of these two diseases in humans, thereby limiting their application (Brouwer et al., 2023; Rafeek et al., 2021). Osowicki et al. established a dependable control human infection model of *Streptococcus pyogenes* pharyngitis (Osowicki et al., 2021). Individuals suffering from pharyngitis exhibited elevated levels of salivary pro-inflammatory cytokines, namely IL-1Ra, Interleukin-6 (IL-6), IFNγ, and IP-10. These cytokines were associated with an elevation in the quantity of monocytes and dendritic cells present in the blood, a reduction in the number of conventional CD4^+^ T cells and B cells, and an elevation in the expression of γδ T-cell activation markers. Additionally, mucosa-associated invariant T (MAIT) cells merit attention due to their capacity to recognize MR1 antigens produced during microbial riboflavin biosynthesis, thereby eliciting protective innate immune responses against microorganisms that generate such metabolites. Emgård et al. Identified MAIT cells contribute to the initial cytokine response to GAS. Studies have also shown high activation of human MAIT cells in patients with streptococcal toxic shock syndrome, and that removal of MAIT cells can reduce the production of cytokines such as IFNγ, IL-1β, IL-2, and TNF (Emgård et al., 2019; Guilherme et al., 2004). The involvement of MAIT cells in the pathogenesis of pharyngitis, invasive GAS disease, and ARF is well-established (Brouwer et al., 2023; Guilherme et al., 2004). Furthermore, infection with GAS, which also impacts the patient’s immune system, is responsible for ARF. Kim et al. systematically identified the immunological mechanisms responsible for the pathogenesis of both diseases, specifically with regard to the interleukin-1β-granulocyte-macrophage colony-stimulating factor (IL-1β-GM-CSF) axis (Kim et al., 2018). Individuals with ARF show continuous production of interleukin-1β in peripheral blood mononuclear cells. This can lead to dysregulated cytokine signaling, specifically the IL-1β-GM-CSF axis, and may be a risk factor for the development of both diseases. Dysregulated cytokine signaling should be considered when evaluating the pathophysiology of ARF, and may provide an explanation for the specialized migration of TH_1_ cells to the mitral valve of the heart; it may also provide an avenue for the advancement of immunomodulatory drugs aimed at treating patients at high risk for ARF (Kim et al., 2018; Lukens et al., 2012). The main source of GM-CSF is CD4^+^ T cells, which are also involved in the pathogenesis of myocarditis by promoting the development and survival of T_h_ cells, as well as their role in autoimmunity and inflammation (Noster et al., 2014). Moreover, it is plausible that NLRP3 Inflammasome may contribute to the pathogenesis of RHD via the induction of pro-inflammatory cytokines, such as IL-1β and IL-18, which in turn stimulate CD4^+^ T lymphocytes and the subsequent release of associated inflammatory cytokines (Rani and Toor, 2023; Kong et al., 2022). In addition, Faé et al. discoveried CXCL9/Mig can enhance T-cell recruitment in valvular tissue lesions, which is linked to the progression of ARF toward RHD (Brouwer et al., 2023; Faé et al., 2013). Recent studies have revealed the important role of C-C chemokine receptor type 2-positive (CCR2+) macrophages in autoimmune diseases and tissue fibrosis, and suggest that they are critical for valve remodeling in RHD (Zhu et al., 2021; Bai et al., 2024). M1 macrophages and the very late antigen 4 (VLA4)/vascular cell adhesion molecule-1 (VCAM-1) pathway may be involved in the inflammatory infiltration and valvular fibrosis in RHD (Xian et al., 2024). The upregulation of VCAM-1 on the surface of endothelial cells represents a key initiating event in the pathogenesis of valve injury, oedema and T-cell infiltration. This leaves the valve vulnerable to immune-mediated damage (Cunningham, 2012; Guilherme et al., 2004; Roberts et al., 2001). In addition, there is a hypothesis that initial infection with GAS may trigger the activation of inflammatory mediators such as TNF-α and IL-6, resulting in endothelial and mesenchymal cells of the heart valves undergoing epigenetic modifications. This modification involves upregulation of the expression of genes that promote cell necrosis and inflammatory responses, including those involved in the TGF-β_1_ signaling pathway. In the pathogenesis of RHD, TGF-β_1_ signaling may cause increased valve fibrosis (Kirvan et al., 2022; Karthikeyan et al., 2020).With recurrent streptococcal infections, the accumulation of IgG_2_ antibodies may activate the inflammatory response in the valve endothelium and promote the attack of inflammatory T-cell subsets such as Th_1_ and Th_17_ on the valve endothelium (Kirvan et al., 2022). In addition, the correlation between ARF and GAS infection may be attributed to TGF-β_1_ overreactivity during mucosal infections, which may enhance Th_17_ cell activity and impede the proliferation of regulatory T cells (Dileepan et al., 2016). Furthermore, there is growing evidence that the microbiome and the immune system may also affect the development of RHD. Dysbiosis may lead to a breakdown of immune tolerance in the host, followed by systemic immune dysregulation, ultimately favoring a pro-inflammatory response to RHD (Nagarajan et al., 2023). These novel findings provide insights that will aid in gaining a comprehensive understanding of the pathogenesis of ARF and RHD.

## GAS vaccine development

The development of vaccines against GAS infections to prevent the disease processes of RF and RHD remains an unresolved challenge in the field of medicine. Since the 20th century, research into GAS vaccines has encompassed M protein–based subunit vaccines, other peptide vaccines, and non-M protein vaccines (**Table 3**). Despite the advent of peptide subunit vaccines representing the latest trend in vaccine research, there is currently no commercially available GAS vaccine (Fan et al., 2024; Ambari et al., 2024; Castro and Dorfmueller, 2021). It is therefore imperative that a global effort be made to develop a widely applicable, effective, and safe GAS vaccine to prevent GAS infection.

**Table 3:** GAS vaccine candidates.

GAS vaccine	Phases	Vaccine candidate		Ref.
M protein vaccine candidates	Preclinical phases	N-terminal peptide-based subunit vaccines	Mx10, 26-valent vaccine	(Fan et al., 2024; Wang et al., 2023; Ambari et al., 2024; Mills et al., 2020; Loh et al., 2021)
		Conserved C-repeat region peptide-based subunit vaccines	StreptInCor,Synthetic P*17 epitope,Synthetic J8 epitope,Synthetic J8 epitope,Synthetic J8 peptide sequence,Synthetic J14 epitope	
	Clinical phases	Hexavalent amino-terminal M protein polypeptide, StreptAvax,MJ8VAX,StreptAnova		
Non-M protein vaccine candidates	Preclinical phases	Single non-M protein subunit vaccine	Group A carbohydrate (GAC), A transpeptidase Sortase A, C5a peptidase,Streptococcal pyrogenic exotoxin A/B, Streptococcal pili,Calprotectin	
		Multicomponent non-M protein subunit vaccines	C5a peptidase-conjugates, GAC-arginine deiminase (ADI), Combo#4,Combo#5, Spy7,SPy_2191,5CP,VAX-A1,TeeVax	

## Summary

ARF and RHD are known to be triggered by recurrent GAS infections. Molecular mimicry is now recognized as an important mechanism in the pathogenesis of ARF. Studies have revealed the presence of anti-collagen antibodies in RHD, as well as anti-cardiac myosin antibodies. Nonetheless, there is still a dearth of a pathogenetic mechanism for fully elucidating ARF, and a comprehensive investigation into the pathogenesis of ARF and RHF is imperative to enhance the prevention and management of these diseases.
